# Iatrogenic Cushing’s Syndrome Following Short-Term Intranasal Steroid Use

**DOI:** 10.4274/Jcrpe.726

**Published:** 2012-09-11

**Authors:** Deep Dutta, Shivaprasad KS, Sujoy Ghosh, Satinath Mukhopadhyay, Subhankar Chowdhury

**Affiliations:** 1 IPGMER & SSKM Hospital, Department of Endocrinology & Metabolism, Kolkata, India

**Keywords:** Cushing’s syndrome, eye drops, dexamethasone, epistaxis

## Abstract

Cushing’s syndrome (CS) is common after oral steroid use and has also been reported following topical or inhaled use, but it is extremely uncommon after intranasal administration. In this paper, we present the case of a child who developed CS after intranasal application of combined moxifloxacin-dexamethasone eye drops for epistaxis for a period of 3 months. CS caused by ocular preparations of steroids has not been reported previously. This case report highlights the fact that even eye drops can contain high doses of steroids and can lead to CS especially in children and especially if used intranasally. Ocular steroid drops should not be used intranasally. To minimize gastrointestinal absorption and therefore the risk of CS, nasal sprays should be preferred over nasal drops for intranasal steroid application.Key words: Cushing’s syndrome, eye drops, dexamethasone, epistaxis

**Conflict of interest:**None declared.

## INTRODUCTION

Development of iatrogenic Cushing’s syndrome (CS) due to oral steroid use is common and is dependent on the dose and the potency of the steroid as well as on the duration of the treatment. Some features of CS like increased intraocular pressure, cataract, benign intracranial hypertension, aseptic necrosis of the femoral head, osteoporosis, and pancreatitis are more frequently observed after exogenous steroid exposure (1). Topical steroid use has occasionally been associated with CS, and in a recent review, it was stated that 43 cases had been reported in the past 35 years, mainly from developing countries (2). Most of these cases were infants with diaper rash or adults with psoriasis necessitating long-term topical steroid use (2). CS following inhaled or nasal steroids is rare and has been mainly reported in AIDS patients on ritonavir (a potent inhibitor of hepatic cytochrome P450) who were simultaneously taking inhaled steroids such as fluticasone for reactive airway disease (3,4). CS following use of ocular administration of steroids (eye drops) has not been reported.

Herein, we present the case of a child who developed iatrogenic CS following intranasal use of dexamethasone eye drops for epistaxis. 

## CASE REPORT

A 6-year-old girl was referred to our endocrinology clinic for evaluation. Her height was 102 cm (3^rd^ percentile), weight 18kg (50-75^th^ percentile), and body mass index was 17.3kg/m^2^ (85-97^th^ percentile). Examination revealed facial puffiness (moon facies), hypertrichosis over forehead and back, and acanthosis (Figure 1A and 1B). Striae, edema and proximal muscle weakness were absent. Blood pressure was normal for age. On further enquiry, it was found that the child was prescribed combined moxifloxacin-dexamethasone eye drops (Milflox-DM eye drops, Unimed, India) by her family physician, to be applied intranasally for epistaxis. She had finished 6 bottles of the drops in the past 3 months. Hemogram, serum electrolytes, liver function tests and urine examination were all normal. Hormonal analysis performed on a blood sample taken at 8 am and repeated on 2 consecutive days revealed a low cortisol (<1μg/dL) level as well as a low adrenocorticotropic hormone-stimulated cortisol level (Table 1). 

The steroid drop administration was stopped; hydrocortisone was started in a dose of 15mg/day (10 mg in the morning after waking up, half an hour before getting out of the bed; 5 mg at 3 pm). The dose was tapered to 10mg/day in the morning only after 1 week. The patient was followed closely to detect any signs of illness or stress. Calcium supplementation was also started. When the patient was evaluated 1 month after the initial diagnosis, it was observed that a significant decrease in the facial puffiness had occurred.

## DISCUSSION

CS following use of intranasal steroids is rare (5,6,7). Perry et al (4) presented a series of 9 children with CS following use of steroid nasal drops (betamethasone, beclomethasone, fluticasone and flunisolide) for ear, nose and throat (ENT) problems. These children were either asymptomatic for any endocrine disorder at presentation or presented with growth failure or features of CS. The duration of steroid use in this series ranged from 0.7 to 6.3 years with a mean of 2.9 years (5). Growth retardation, hypopituitarism, osteoporosis and hypertension were also reported in a 19-year-old boy who took intranasal dexamethasone for more than 5 years, for nasal obstruction, which reversed after tapering off and stopping the steroids (6). CS following intranasal administration of dexamethasone for allergic rhinitis has also been reported (7).

Our patient presented with CS after only a 3-month use of steroid drops. She was prescribed combined moxifloxacin-dexamethasone eye drops for epistaxis (Milflox-DM, Unimed, India; 10 mL bottle), a solution containing 1 mg/mL of dexamethasone phosphate, i.e. 10 mg of dexamethasone per bottle. Her use of 2 bottles per month is equivalent to 20 mg of dexamethasone per month which is around 0.7mg of dexamethasone per day, which is high for a 6-year-old girl. Moxifloxacin does not inhibit or stimulate the hepatic microsomal P450 system and hence has no effect on steroid metabolism (8). Since drops were used instead of nasal spray, it is likely that a significant amount would be swallowed and absorbed from the gastrointestinal tract, resulting in CS. The normal daily cortisol production in our body ranges from 8 to 15 mg/day (9). Hence, our patient was shifted to hydrocortisone of 15 mg/day then tapered to 10 mg/day. Hydrocortisone was used as it is the most physiological preparation of steroid commercially available, causes least hypothalamic-pituitary-adrenal (HPA) axis suppression and is most suitable for allowing the HPA axis to recover. 

The purpose of presenting this case was to highlight the occurrence of CS even with steroid eye drop preparations, some of which can contain high concentrations of steroids. Long-acting steroids with a high potency, such as dexamethasone, increase the risk of developing CS. Ocular steroid preparations should not be administered by other routes. Intranasal sprays should be preferred over drops for intranasal administration of steroids to decrease the risk of swallowing and thus of gastrointestinal absorption. Regular monitoring of height and weight is necessary in children on intranasal steroids and periodic estimation of morning cortisol level may be done in these children to evaluate the status of the HPA axis. Exogenous steroid use (oral, topical, inhaled or intranasal) should always be taken into consideration in a child with weight gain or height arrest, as the diagnosis of iatrogenic CS is often missed if not enquired thoroughly when taking the patient’s history. 

In conclusion, iatrogenic CS with suppressed HPA axis developed in a child following intranasal use of dexamethasone eye drops. The condition improved after stopping the treatment and shifting the patient to gradual tapering of hydrocortisone.

## Figures and Tables

**Table 1:**
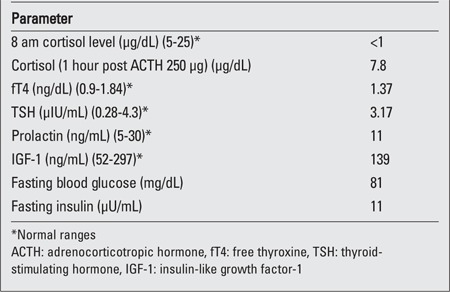
Hormonal profile of the patient

**Figure 1a:**
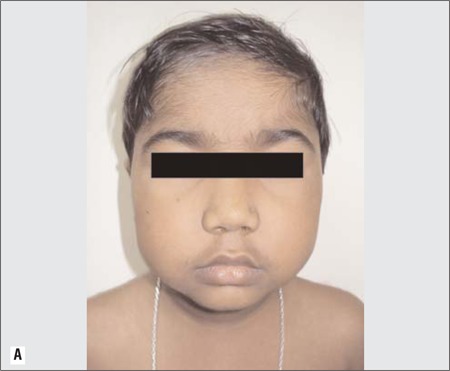
A) Moon facies with hypertrichosis over forehead and lips

**Figure 1b:**
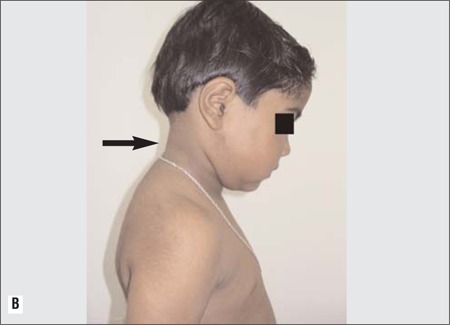
B) Profile of child showing acanthosis (black arrow) around the neck
